# Partition-Based Joint Placement of Gateway and Controller in SDN-Enabled Integrated Satellite-Terrestrial Networks

**DOI:** 10.3390/s19122774

**Published:** 2019-06-20

**Authors:** Kongzhe Yang, Bangning Zhang, Daoxing Guo

**Affiliations:** College of Communication Engineering, Army Engineering University of PLA, Nanjing 210007, China; kongzheyang@gmail.com (K.Y.); dxgguo@sina.cn (D.G.)

**Keywords:** Integrated Satellite-Terrestrial Networks (ISTNs), Software-Defined Networking (SDN), joint gateway and controller placement, propagation latency, network reliability

## Abstract

The Integrated Satellite-Terrestrial Networks (ISTNs) with Software-Defined Networking (SDN) incorporated have become the emerging architecture and have attracted considerable attention recently. Therein, the joint gateway and controller placement problem is of fundamental significance for designing ISTNs with flexible and efficient management capabilities. Hence, how to achieve network reliability maximization with low network latency in the joint placement problem is worthy of in-depth study. In this paper, a network partition algorithm, Simulated Annealing Partition-based K-means (SAPKM), is proposed and analyzed for further ameliorating the problem. Experiments are performed on real network topologies to validate the effectiveness of our approach for the joint placement problem. Compared with the state-of-the-art existing works, numerical results show that SAPKM outperforms when deploying four or more controllers in terms of network reliability performance, network latency, and inter-plane latency with less than 2 ms to converge.

## 1. Introduction

Recently, many of the latest technical reports published by 3GPP have shown the significance of incorporating satellite networks into 5G scenarios [1,2]. Satellite communication networks will be a complementary solution to satisfy multiple performance metrics in the 5G system, with the merits of ubiquitous coverage and broadcast capability to improve the reliability and availability of the overall network. Expected to enlarge the coverage beyond terrestrial networks, the integrated satellite-terrestrial networks (ISTNs) are effective at providing resilient and high-reliability service for rural, remote, and urban areas, particularly for battlefields with critical missions [3,4,5,6]. Compared with traditional terrestrial or satellite networks, the ISTNs are subject to limited and unbalanced network resources [7,8,9]. How to explore the new network architecture to supply services and applications in different scenarios with various Quality of Service/Experience (QoS/QoE) requirements is challenging [10,11,12]. Thus, the system integration [13], protocol optimization [14], resource allocation [15], and network management [16] in ISTNs are of great significance.

New paradigms like Software-Defined Networking (SDN) and Network Function Virtualization (NFV) not only facilitate the seamless integration, but also reduce both operational and capital expenses of ISTNs. SDN/NFV features can provide significant benefits over satellite networks [17,18] and terrestrial ones [19,20]. Previous works have proven the efficacy of the adoption of SDN and NFV technologies into the satellite domain [21] and terrestrial domain [22], which seems to be a necessary step to promote the integrated network. Moreover, the incorporation of SDN/NFV technologies facilitates the integration of satellite communications networks with the 5G terrestrial ones. However, some remaining unsolved problems, related to further exploiting the potentialities of SDN approaches for the integration, are worthy of in-depth study, especially for adaptive and flexible management in dynamic environments.

The separation of the control plane and data plane as the distinctive feature of SDN makes it possible to manage the entire integrated network intelligently. Specifically, a set of dedicated controllers composing the control plane manages devices in the data plane, including switches and satellite gateways, to forward packets or to execute the handover flexibly and efficiently. Apart from the latency depending on the placement of both gateways and controllers in certain topologies, reliability is another vital performance indicator influenced by the joint placement. Since the joint placement of both satellite gateways and controllers has an influence on the network performance, how to determine the number and the locations of gateways and controllers needs further investigation.

In this paper, we propose an efficient network partition algorithm to tackle the joint gateway and controller placement problem in the SDN-enabled ISTNs. In the proposed algorithm, the ground segment of the entire networks is divided into multiple sub-domains, and only one controller is deployed in each of them. By incorporating the partition-based K-means algorithm [23], the average latency between the centroid and other nodes in each sub-domain can be promptly shortened. Hence, both the performance and running time are notably optimized compared with previous joint placement algorithms [24].

Moreover, we further decrease the algorithm complexity with better performance on network reliability and controller-switch propagation latency. On the one hand, the merits of the partition strategy on shortening the distance among the nodes within the sub-domains decrease the network propagation latency. On the other hand, other performance objectives, including load balancing and consistency, can be implemented in the sub-domains instead of the whole network, which increasingly reduces the complexity of the algorithm.

We evaluate the average network latency, the average network reliability, the average/maximum controller-switch latency, and the running time of our proposed algorithms on real network topologies of various kinds, and the results are compared with the existing SACA method [24]. The main contributions of this paper are briefly highlighted as follows.

- A Simulated Annealing Partition-based K-Means (SAPKM) algorithm is proposed to ameliorate the joint placement problem in the SDN-enabled ISTNs. By adopting SAPKM, performance indicators can be accomplished in the sub-domains instead of the whole network, which dramatically reduces the complexity of the joint placement problem. Besides, selecting the centroids of the sub-domains as initialized nodes further decreases the number of redundant iterations.
- The joint placement problem in terms of reliability maximization with network latency constraints is analytically formulated. With the appropriate deployment for gateways and controllers, the proposed algorithm will shed light on other performance metrics including controller-switch latency, load balancing, and even the trade-offs among multiple objectives.
- Considering the intrinsic properties of network topologies, i.e. the structure and the density of the internal nodes, experiments are performed on real network topologies varying in size and structure.
- Compared with the existing algorithms, simulation results indicate the merits of the proposed algorithm in shortening network latency, enhancing network reliability with much lower complexity, especially for adapting to large topologies with multiple gateways and controllers to deploy. All the adaptive traits are propitious for studying the online joint placement problem for dynamic networks in the future.

The rest of the paper is organized as follows: Section 2 presents the related works in recent years. Section 3 introduces the general architecture of the SDN-enabled integrated satellite-terrestrial networks and formulates the joint gateway and controller placement problem mathematically with constraints. Section 4 introduces the proposed algorithm SAPKM and analyzes the complexity of it. Section 5 presents the numerical results, which show the superiority of SAPKM in both performance and complexity compared with previous works. Finally, concluding remarks are drawn in Section 6.

## 2. Related Works

Taleb et al. [3] first highlighted the opportunities behind the ISTNs and discussed the potentials and solutions of the interworking operations between the satellite and terrestrial domains. Subsequent studies analyzed the challenges of satisfying the QoS requirements for seamless and converged ISTNs [4,25]. Bertaux et al. [26] investigated the advantages of incorporating network programmability and virtualization using SDN/NFV in satellite networks and demonstrated that integrating these approaches will benefit communication services over satellite networks. Besides, Yang et al. [27] proposed an SDN-based seamless handover mechanism to provide flexible broadband satellite services for satisfying diverse QoS requirements over satellite networks. Then, Evans [28] addressed the role of satellites in 5G and summarized key challenges of the ISTNs towards 5G. In terms of the heterogeneous network convergence aspects, Feng et al. [5] proposed an SDN/NFV-based flexible network architecture, HetNet, to enable efficient integration of ISTNs.

When it comes to the control plane of SDN, Heller et al. [29] first proposed the Controller Placement Problem (CPP) to determine the number and locations of required controllers, which directly affects the entire network performance. Considering the heterogeneity and interconnections of controllers, Sallahi et al. [30] studied the optimal model for CPP, which was merely practical for the small-scale networks. Heuristic analysis was adopted in [31] to addresses the controller placement problem with respect to various important metrics, including latency, resilience, and load balancing.

Subsequent works concentrated on various performance metrics, such as reliability [32], controller capacity [33], latency [34], load balancing [22], and efficient placement algorithms [35]. In particular, Hu et al. [32] not only proved the NP-hardness of the reliability-aware controller placement problem, but also proved the effectiveness of the Simulated Annealing Algorithm (SAA). Besides, Ros et al. [36] firstly showed that the network reliability was principally determined by the network topology structure, especially the density of the internal nodes. The authors analyzed the impact of controller number on reliability, as well as the trade-off between reliability and latency.

Considering the reliability and capacity of controllers, another work [34] proposed a controller placement strategy planning ahead for controller failures to make the networks more reliable and more resilient. Furthermore, high-quality and cost-saving solutions for resilient capacitated CPP were derived from [22], which took both the switch-controller/inter-controller latency and the capacity of the controllers into consideration. In addition, the authors in [37] addressed the dynamic CPP, which took the dynamic traffic load of switches into account to bound the communication latency.

Meanwhile, since data in the integrated networks is transmitted from switches to satellites via satellite gateways, the Gateway Placement Problem (GPP) also affects the network performance. Previous works [38,39] studied GPP in ISTNs to maximize network reliability with capacitated gateways and a propagation latency constraint.

Available studies showed the recent progress of CPP or GPP in terrestrial networks, without considering the joint gateway and controller placement simultaneously. Liu et al. [24] was the first work to provide a detailed overview of the joint placement of controllers and gateways in ISTNs. Moreover, it also proved that the joint placement problem is a multi-object problem, which is totally distinguished from either CPP or GPP in terrestrial networks.

Unlike the above-mentioned papers, we took the intrinsic properties of the given topologies into account, i.e., the structure and the density of the internal nodes, and hybridized the network partition scheme to solve the joint placement problem. Various real network topologies with different properties were employed to demonstrate the superiority of the proposed algorithm, with better performance and less complexity.

## 3. System Model

In this section, the system model of the SDN-enabled integrated satellite-terrestrial networks is illustrated at first. Then, we model the joint placement problem as a Mixed Integer Linear Program (MILP) with network latency and network reliability formulated mathematically.

### 3.1. An Architecture for SDN-Enabled Integrated Satellite-Terrestrial Networks

**Figure 1:**
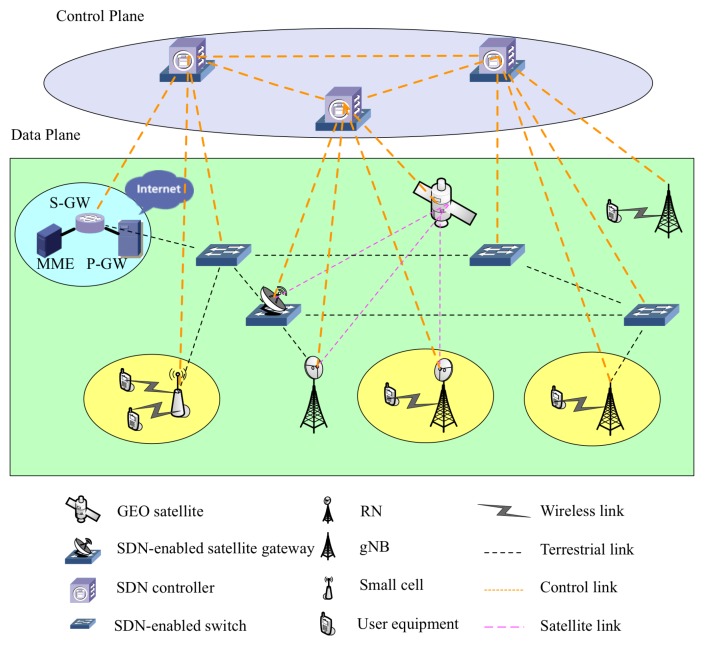
An architecture for SDN-enabled integrated satellite-terrestrial networks.

In this subsection, an SDN-enabled ISTN model is briefly introduced. As illustrated in Figure 1, the considered ISTN consisted of two logical parts, the data plane and the control plane. In the control plane, numerous SDN controllers hosted on the physical nodes of the data plane provided logically-centralized control and management functions. On the other hand, in the data plane, the user equipment of all types connected to the terrestrial networks through the gNBs (gNodeBs) and the RNs (Relay Nodes). The terrestrial networks mainly relied on optical fiber to establish connections between the SDN nodes in the backhaul, as well as the Radio Access Network (RAN) and core network. Besides, a high throughput Geostationary orbit (GEO) satellite was set as a complementary part of the terrestrial networks in the model. It is noted that satellite communications with the ground nodes were implemented through the satellite gateways and RNs.

Since the main interest was optimizing the joint gateway and controller placement to maximize the network reliability with network latency constraints, the analysis was simplified, and neither the core network nor RAN were considered in the proposed architecture.

### 3.2. Preliminary Assumptions

In the previous subsection, we introduced the transmission process of the considered ISTN; thus, here we focus on the leading culprits in transmission latency and network reliability.

Assuming a controller or a gateway can be placed at the locations of switches separately, the ultimate goal is to place an appropriate number of gateways and controllers to maximize the network reliability with latency constraints.

Distinguished from traditional networks, a significant feature of SDN is the separation between the control plane and the data plane. Specifically, the control plane consists of multiple dedicated controllers, while the data plane is composed of multiple switches and gateways for forwarding packets. Thus, in the SDN-enabled integrated networks, the distributed controllers located on the ground facilitate network control and network services. We assumed that the satellite was also identified as an SDN switch to receive control instructions from the controllers.

In pure SDN-enabled networks, we are mainly concerned with the placement of controllers and assignment with switches, while in SDN-enabled networks, the joint placement of gateway and controller affect many performance metrics. In other words, in addition to the controller-switch path, another two kinds of paths, i.e., gateway-switch and controller-gateway, should also be taken into consideration in SDN-enabled ISTNs. The relationship between the different kinds of paths and the performance metrics are illustrated in Table 1.

Apparently, in pure SDN-enabled networks, there are only two kinds of nodes, the controller and the switch, in the network topology. Thus, the placement of the controllers and the assignment with switches determine the network reliability and the inter-plane latency. However, in SDN-enabled ISTNs, we mainly are concerned with the latency between the satellite and the user equipment on the ground through the gateways. Since the distance between the GEO satellite and the gateways on the surface of the Earth can be assumed as constant, while the distance between the gateways and the switches varies from according to the placement and the assignment between them, hence we focused on the process of transmission on the ground and defined the latency between the gateways and the switches as the network latency. That is to say, the gateway-switch paths determine the network latency, which is the difference between the pure SDN-enabled networks and the SDN-enabled ISTNs.

The network latency between switches and the satellite consists mainly of two parts: propagation latency and forwarding latency. The former is mainly determined by the distance between switches and the satellite, while the latter is basically affected by the load of the nodes. Due to the huge coverage of the GEO satellite, the ground segment of the SDN-enabled ISTNs can be regarded as the Wide area Network (WAN), where numerous works have studied the controller placement problem. Here, we mainly study the effect of the propagation latency on the joint placement problem owing to the relative static assignment between gateways and switches in the architecture. Therefore, how to minimize the average propagation latency of the integrated networks is worthy of in-depth study, which depends on the number and locations of gateways.

Since any node or link failure on occasion may disconnect the control plane and the data plane, invalid paths due to nodes’ or links; failure may result in severe packet loss and performance degradation. Consequently, how to place properly gateways and controllers simultaneously to limit the impact of failed links and nodes on network reliability is of utmost importance.

It should be noted that we here focused on the appropriate placement of various numbers of gateways and controllers; the optimum number of gateways and controllers and the traffic delivery between the control plane and the data plane are beyond the scope of this paper. Moreover, considering the dominance of propagation latency in transmission latency between controllers and switches, the controller-switch latency should also be taken into account in the joint placement problem.

### 3.3. Joint Placement Metrics

In the previous subsection, we briefly introduced the joint placement problem for network reliability maximization with latency constraints in SDN-enabled ISTNs. Here, we formulate the problem and the performance metrics mathematically.

For any switch $u\in U,L_{uw}^{s}$ denotes the latency of the path from switch *u* to the satellite *s*, via gateway *w*, which can be defined as:

(1) $$L_{uw}^{s}=L_{uw}+L_{sw},$$

where $L_{uw}$ and $L_{sw}$ denote the latency from switch *u* to gateway *w* and the latency from *w* to the satellite *s*, respectively. Since the distance between the GEO satellite and the ground can be considered as fixed while all data traffic transmission between the switches and the satellite has to pass through gateways, the latency between switches and gateways plays a principal role. Thus, the average network latency can be formulated as:

(2) $$L_{avg}=\frac{1}{n}\sum_{\begin{array}{c} u\in U \end{array}}L_{uw},\;w\in W.$$

Once the satellite gateway placement problem is established, the average network latency of the integrated networks will be determined.

Besides, the average and maximum propagation controller-switch latency can be defined respectively as:

(3) $$L_{uc,\mathrm{avg}}=\frac{1}{n}\sum_{\begin{array}{c} u\in U,c\in C \end{array}}L_{uc},$$

(4) $$L_{uc,\max}=\max_{\begin{array}{c} u\in U,c\in C \end{array}}L_{uc}.$$

Owing to the influence of geographical locations and the distribution of the nodes, different placements of controllers can lead to different network reliability performances, while different placements of gateways influence both network latency and reliability to varying degrees.

For the set of terrestrial nodes U, we defined $P_{u}$, $P_{e}$, and $P_{e_{sw}}$ as the failure probability of ground node *u*, terrestrial link *e*, and satellite link $e_{sw}$ between satellite *s* and gateway *w*, respectively. According to previous works [29,32], the shortest path from *u* (or *s*) to *c* was selected by using the Dijkstra algorithm. Here, we divided the reliability of the path into two types: $R_{uc}$ is defined as the reliability of the path from switch *u* to controller *c*, and $R_{wc}^{s}$ is defined as that from satellite *s* to controller *c*, via gateway *w*. Let $U_{u\to c}$ denote the node set and $E_{u\to c}$ denote the link set on the path from u∈U to c∈C. $R_{uc}$ can be calculated as:

(5) $$R_{uc}=\prod_{e\in E_{u\to c}}\left(1-P_{e}\right)\prod_{u\in U_{u\to c}}\left(1-P_{u}\right),$$

Similarly, $R_{wc}^{s}$ is formulated as:

(6) $$R_{wc}^{s}=\left(1-P_{e_{sw}}\right)\prod_{e\in E_{s\to c}}\left(1-P_{e}\right)\prod_{u\in U_{s\to c}}\left(1-P_{u}\right).$$

Hence, the average reliability of the integrated networks is defined as:

(7) $$R_{avg}=\frac{1}{n+k}\left(\sum_{\begin{array}{c} u\in U \end{array}}R_{uc}+\sum_{\begin{array}{c} s \end{array}}R_{wc}^{s}\right),\;c\in C,w\in W,$$

where *n* and *k* represent the number of switches and gateways, respectively, and C represents a set of controllers.

### 3.4. Problem Formulation

Thus, the joint gateway and controller placement problem can be formally defined as follows. Given the GEO satellite *s* and the set of ground nodes U with *k* satellite gateways and *m* SDN controllers for deployment, we aim at determining the optimal placement of gateway $W=\{w_{1},w_{2},\ldots ,w_{k}\}\subseteq U$ and controller $C=\{c_{1},c_{2},\ldots ,c_{m}\}\subseteq U/W$, so as to maximize the average network reliability with $L_{\mathrm{cst}}$ as the network latency constraint, namely,

(8) $$\max_{\begin{array}{c} c\in C,w\in W \end{array}}\;\;R_{avg},$$

(9) $$s.t.\;\;\;L_{avg}\leq L_{\mathrm{cst}},\;w\in W,$$

(10) $$\sum_{j\in C}c_{j}=m,$$

(11) $$\sum_{j\in C}u_{ij}=1,\;\;\;\forall i\in U,$$

(12) $$u_{ij}\leq c_{j},\;\;\;\forall i\in U,j\in C$$

(13) $$\;\sum_{j\in C}w_{lj}=1,\;\;\;\forall l\in W,$$

(14) $$w_{lj}\leq c_{j},\;\;\;\forall l\in W,j\in C$$

(15) $$u_{ij},w_{lj},c_{j}\in \left\{0,1\right\},\;\;\;\forall i\in U,l\in W,j\in C$$

The constraints of this MILP model can be divided into three categories: latency-related, placement-related, and numeric-related. Among them, Equation (9) is latency-related, which means that the average network propagation latency should meet the latency constraints. Besides, the ensuing constraint Equations (10)–(14) are placement-related where Equation (10) means there are exactly *m* controllers deployed separately in the network; Equation (11) means that each switch is assigned to one controller; Equation (12) means there is no control path between node *i* and *j* without a controller placed at node *j*; Equations (13) and (14) mean similar constraints on gateways; finally, Equation (15) is the numeric constraints.

However, finding the joint placement solution with maximized reliability is NP-hard, which means there is likely no polynomial-time algorithm that can guarantee an optimal solution. Therefore, we considered a heuristic approach by incorporating the network partition scheme to ameliorate the problem.

## 4. Network Partition Algorithm for the Joint Placement Problem

In this section, a partition-based algorithm is adopted to handle the joint placement problem. The given topologies will be divided into multiple sub-domains to simplify the deployment process. It is worth mentioning that selecting the centroids as the initial sets contributes to further reducing the complexity of the problem.

We assumed the SDN-enabled ISTN was modeled as an undirected graph G=(V,E), where V represents the set of nodes and E the set of physical links among them, where the link weight refers to physical distance. Detailed notations and definitions are summarized in Table 2.

### 4.1. Simulated Annealing Partition-Based K-Means Algorithm

By incorporating the network partition scheme, two partition-based algorithms are proposed to address the gateway placement problem and joint placement problem in this subsection, respectively.

The core of the network partition is to separate the integrated networks into multiple sub-domains by using appropriate methods. In the standard K-means algorithm, the initial nodes are randomly selected, and the network partition varies from each iteration. Meanwhile, the main idea of SAA is to iterate a new neighbor solution every execution to optimize the objective function continuously, with a random initialization.

There are two kinds of nodes in the ground segment of the network topology, i.e., satellite gateways and SDN controllers. In order to obtain available joint placement solutions with the maximized network reliability under latency constraints, we should partition the network in terms of the gateways and the controllers, respectively, while previous works were only concerned with the network partition in terms of the controllers. In other words, not only the sub-domains for gateways and controllers, but also the purposes of the partitions were different. The partition results in terms of the gateways were *k* sub-domains with all available nodes, while those for the controllers were *m* sub-domains with all the nodes except the ones occupied by the gateways. The purpose of the gateway placement was the minimization of network latency, while the purpose of the controller placement in the second phase was the maximization of network reliability.

For solving the satellite gateway placement problem, a simple solution is to enumerate all possible combinations of *k* satellite gateways of *n* nodes in U and then choose an optimal solution that achieves the minimum average network latency. However, due to its exponential computational complexity, this Optimal Enumeration Algorithm (OEA) has to run for a very long time, especially when the number of nodes in a certain topology is large.

Thus, enlightened by the idea of the clustering-based network partition algorithm in [23], we reconsidered the placement problem and proposed the Partition-based K-Means algorithm (PKM) to address GPP, which is summarized in Algorithm 1. The main idea of PKM is to divide the given topology into a certain number of sub-domains by iteratively partitioning the topology and updating the centroids of them.

**Algorithm 1** Partition-based K-Means algorithm (PKM).

**Require:**
  G(V,E): the target topology of a certain physical network.  *k*: the target number of satellite gateways.


**Ensure:**
  $W_{\mathrm{opt}}$: the optimized placement of *k* satellite gateways.  $L_{\mathrm{ave}}$: the average network latency between gateways and switches.

1:Select one node randomly from U as $W_{\mathrm{new}}$ of G.2:Generate the initial $W_{\mathrm{opt}}$ of G.3:**while** the number of sub-domains is less than *k* **do**4: Searching for the next target sub-domain to be partitioned, which has the longest shortest path distance from the internal nodes to its centroid. The node that has the longest distance to its centroid is denoted as the next center$w^{*}$.5: $W_{\mathrm{new}}=W_{\mathrm{new}}\cup \left\{w^{*}\right\}$.6: Generate the updated $W_{\mathrm{opt}}$ by updating the centroid of each sub-domain.7:
**end while**
8:Compute $L_{\mathrm{ave}}$ according to $W_{\mathrm{opt}}$.9:**return**$W_{\mathrm{opt}}$, $L_{\mathrm{ave}}$


Similar to GPP, the Optimal Enumeration Algorithm for the Joint placement problem (OEAJ) is the most intuitive method for obtaining an optimal solution. The authors in [32] proved the effectiveness of SAA in CPP, which is a probabilistic technique for approximating the global optimum of a given function. Here, we propose the Simulated Annealing Partition-based K-Means algorithm (SAPKM) as an efficient method to deal with the joint placement problem. The details of SAPKM are described in Algorithm 2.

Note that the gateway placement has an impact on both network latency and reliability, while the controller placement only affects the network reliability. Therefore, determining the locations of controllers in each sub-domain while fixing the gateways is preferable than determining the locations of the gateways while fixing controllers in the network. In other words, we should first deploy gateways, then consider the deployment for controllers.

As shown in Algorithm 2, the first step is to select two initial sets, $W_{\mathrm{opt}}$ and $C_{\mathrm{opt}}$, which consist of the centroids of the sub-domains separated by the occupied nodes. Then, the network reliability $R_{\max}$ is calculated. At every iteration of the while-loop, new neighbor solutions, $W_{\mathrm{new}}$ and $C_{\mathrm{new}}$, are generated with the updated network reliability $R_{\mathrm{new}}$ obtained. Obviously, the new solution will be accepted if the updated solution’s reliability is larger when satisfying the latency constraints. Besides, the new solution with worse reliability will also be accepted with the acceptance probability $P\left(\Delta \right)=e^{-\frac{\Delta }{T}}$, where $\Delta =R_{\mathrm{new}}-R_{\max}$ and *T* denotes the current temperature.

**Algorithm 2** Simulated Annealing Partition-based K-Means algorithm (SAPKM).

**Require:**
  G(V,E): the target topology of a certain physical network.  *k*: the target number of satellite gateways.  *m*: the target number of SDN controllers.  $L_{\mathrm{cst}}$: the network latency constraint.


**Ensure:**
  $W_{\mathrm{opt}}$: the optimized placement of *k* satellite gateways.  $C_{\mathrm{opt}}$: the optimized placement of *m* SDN controllers.  $L_{\mathrm{ave}}$: the average network latency between gateways and switches.  $R_{\max}$: maximum path reliability of the network.

1:**Initialize**$T=T_{0},T_{\mathrm{final}},\alpha$.2:Partition the topology into *k* sub-domains, and obtain the k−centroids set $W_{\mathrm{ctd}}$.3:Partition the topology into *m* sub-domains, and obtain the m−centroids set $C_{\mathrm{ctd}}$, where $W_{\mathrm{ctd}}\cap C_{\mathrm{ctd}}=⌀$.4:Compute the average reliability $R_{\max}$.5:
**while** $T>T_{\mathrm{final}}$ **do**
6: Generate a new gateway set $W_{\mathrm{new}}$.7: Compute the average network latency $L_{\mathrm{ave}}$.8: **if** $L_{\mathrm{ave}}\leq L_{\mathrm{cst}}$ **then**9:  Partition the topology into *m* sub-domains, and obtain the m−centroids set $C_{\mathrm{new}}$, where $W_{\mathrm{new}}\cap C_{\mathrm{new}}=⌀$.10:  Compute the average reliability $R_{\mathrm{new}}$.11:  $\Delta =R_{\mathrm{new}}-R_{\max}$.12:  Generate a random number δ∈(0,1).13:  **if** Δ≥0 or $e^{\frac{\Delta }{T}}>\delta$ **then**14:   $W_{\mathrm{opt}}=W_{\mathrm{new}}$.15:   $C_{\mathrm{opt}}=C_{\mathrm{new}}$.16:   $R_{\max}=R_{\mathrm{new}}$.17:  **end if**18: **end if**19: T=T·α.20:
**end while**
21:**return**$W_{\mathrm{opt}}$, $C_{\mathrm{opt}}$, $L_{\mathrm{ave}}$, $R_{\max}$


### 4.2. Analysis of the Complexity and Effectiveness of SAPKM

Compared to the state-of-the-art works, SAPKM has the advantage on computational efficiency due to its optimized initialization. The computational complexity of SAPKM is analyzed in detail as follows.

**Theorem** **1.** *The computational complexity of PKM in Algorithm 1 is $O(k^{2}\cdot n)$, where n and k denote the number of nodes and gateways, respectively.*

**Proof.**  The function of PKM in Algorithm 1 is to iteratively obtain *k*-centroids by iteratively updating them from 1 sub-domain till *k* sub-domains.

Obviously, the computational complexity of the process of centroid-based network clustering is O(k·n). Thus, the running time for each iteration of the while-loop in Steps 3–7 is O(n+(k+1)n). Therefore, the running time of Algorithm 1 is $O(\frac{\left(k^{2}+3k-4\right)}{2}\cdot n)$, which can be computed in $O(k^{2}\cdot n)$ time. □

**Theorem** **2.** *Each iteration of the while-loop in SAPKM described in Algorithm 2 can be computed in $O(m^{2}\cdot n+k\cdot n)$ time, where n, k, and m denote the number of nodes, gateways, and controllers, respectively.*

**Proof.**  SAPKM mainly consists of two parts, i.e., initializing the gateways and controllers by employing PKM and updating the joint placement by implementing SAA to maximize reliability with latency constraints. Specifically, Steps 2 and 3 can be computed in $O(k^{2}\cdot n)$ and $O(m^{2}\left(n-k\right))$ time, respectively. For Step 4, the running time is O(k·n+k·m). For Steps 5–20, each iteration of the while-loop runs $\left(k\cdot n+k\cdot m+m^{2}\left(n-k\right)\right)$ times, which can be computed in $O(m^{2}\cdot n+k\cdot n)$ time. □

From the above, Algorithm 2 can be compute in $O(\left(k^{2}+\left(1+t\right)\left(m^{2}+k\right)\right)n)$ time with *t* iterations.

## 5. Simulation Results

The proposed SAPKM formulation was evaluated on various real networks from the Internet Topology Zoo [40], and the comparisons on the performance of the SAPKM and other algorithms, i.e., OEAJ and SACA, were analyzed by simulation. If interested in the details of the proof of the computational complexity of OEAJ and SACA, readers can refer to Theorem 1 and 2 in [24]. Six real network topologies with distinguishing intrinsic properties were included: Nsfnet, Aarnet, ATT, Agis, Geant, and Chinanet, with detailed topologies and failure probability settings listed in Table 3 [24]. In order to obtain stable performance, we repeated random placement algorithms 1000 times and took the average value. All the algorithms were performed in MATLAB (R2018a) running on a MacBook Air, with 1.8-GHz Intel Core i5 CPUs and 8 GB of 1600 MHz DDR3 RAM.

The simulation consisted of the four following steps: First, topology settings were referenced from the Topology Zoo, including the node coordinates and the links among them. Then, the Dijkstra algorithm was employed to choose the shortest path between any two nodes, and the length of the corresponding path was computed by the Haversine formula. Furthermore, the associated network latency was calculated with propagation velocity $c=2\times 10^{8}$ m/s [41]. Finally, with the above look-up topology data and topology settings on failure probability, joint gateway, and controller placement were solved by various efficient algorithms to maximize the system reliability with network latency constraints.

In order to evaluate the performance and complexity of the proposed algorithm, we compared SAPKM with two other representative solutions, OEAJ and SACA. The former is the theoretically best solution to the joint placement problem since all results are enumerated and compared. The latter is an efficient algorithm proposed by [24], which applies the Simulated Annealing and Clustering hybrid Algorithm (SACA) to obtain a near-optimal result solution. It is noted that the authors in [24] proved the superiority of SACA in reliability over other algorithms, i.e., RANDJ, SAKM. Thus, we mainly focused on the comparison results between SAPKM and SACA. We first evaluated the SAPKM algorithm in comparison with the above solutions and then demonstrated how the reliability was enhanced in large topologies with less running time.

### 5.1. Network Latency Minimization

**Figure 2:**
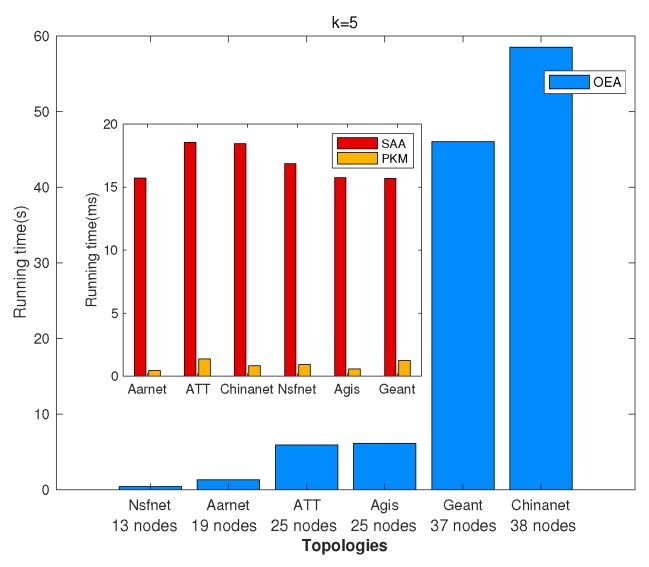
Comparisons of the overall running time.

**Figure 3:**
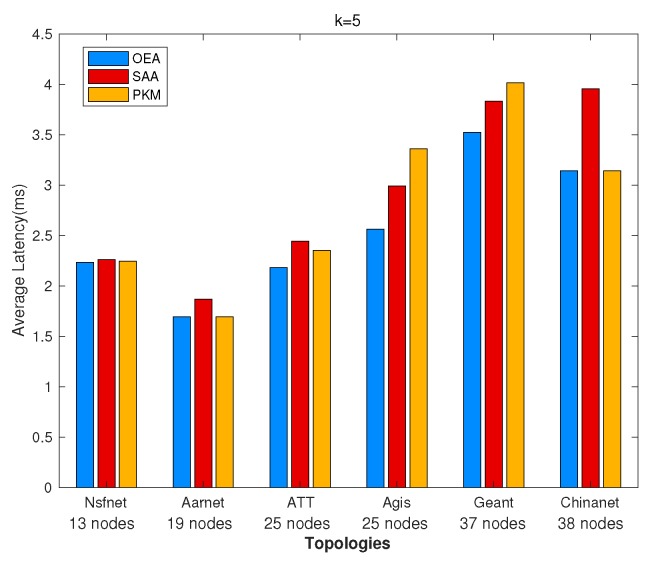
Comparisons of the overall average network latency.

**Figure 4:**
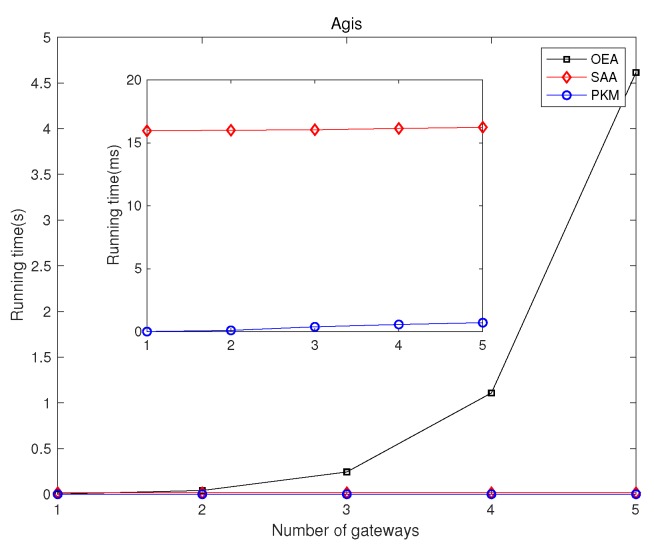
Comparisons of the overall running time in Agis.

**Figure 5:**
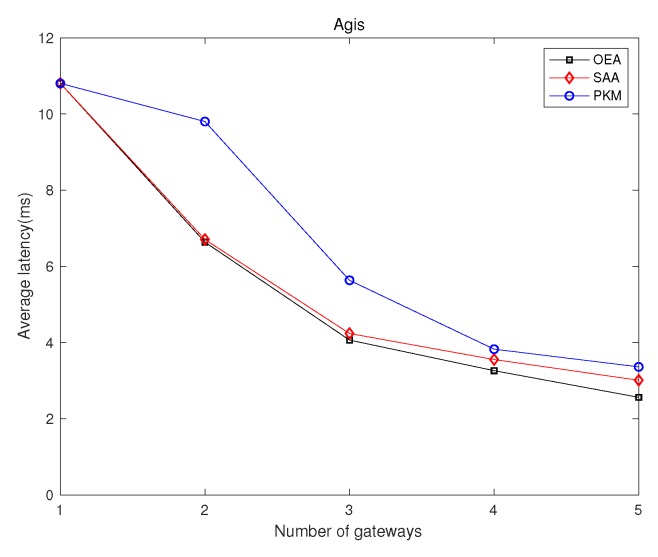
Comparisons of the overall average network latency in Agis.

**Figure 6:**
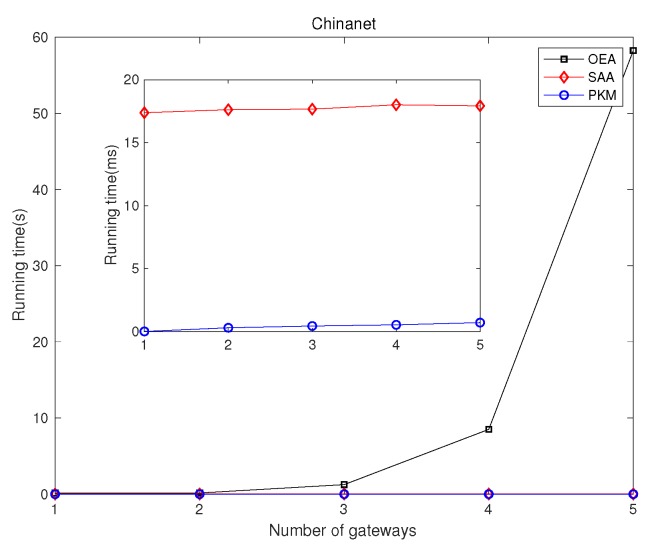
Comparisons of the overall running time in Chinanet.

**Figure 7:**
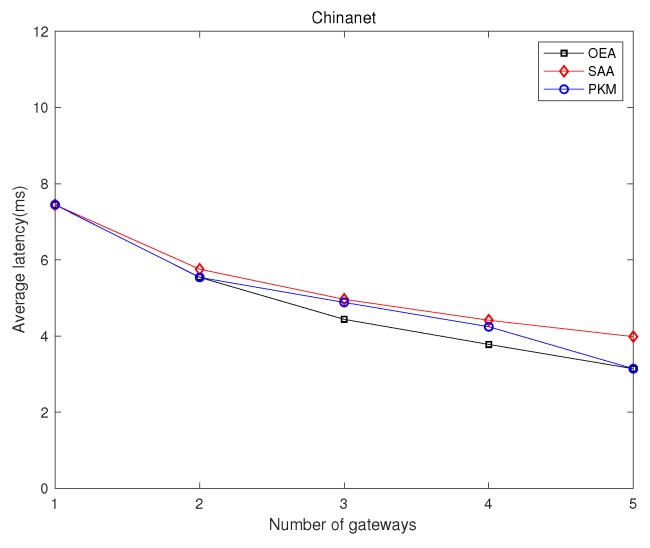
Comparisons of the overall average network latency in Chinanet.

We investigated GPP using different algorithms, i.e., OEA, SAA, and PKM, and made comparisons on the average network latency and running time among them. Figure 2 and Figure 3 depict comparisons of both the overall running time and the overall network latency on the above topologies. Figure 4 and Figure 5 are implemented on the Agis topology (25 nodes), while Figure 6 and Figure 7 are implemented on the Chinanet topology (38 nodes) for further investigation. All the simulations showed the strengths and weaknesses of the above gateway placement algorithms.

Figure 2 demonstrates the comparison results of the overall running time in different topologies when the number of gateways was five. Figure 4 and Figure 6 show the overall running time in Agis and Chinanet when the number of gateways varied from 1–5. It is obviously depicted that PKM and SAA had a short running time, while the running time of OEA increased exponentially as the number of gateways became larger. Besides, the computational complexity of PKM was much lower than that of SAA, with less than 1 ms to converge.

On the other hand, Figure 3 demonstrates the comparison results of average network latency in various topologies when the number of gateways was five. OEA obtained the optimal solution with the lowest average network latency, while both SAA and PKM acquired near-optimal solutions. However, subject to the intrinsic properties of the given topologies, the placement of gateways derived from implementing PKM was fixed. It is noted that the performance, such as network latency and network reliability, depends on characteristics of the topologies. As depicted in Figure 3, PKM showed a better latency performance than that of SAA in Nsfnet, Aarnet, ATT, and Chinanet, where the opposite results occurred in Agis and Geant. Thus, we further investigated the effect of the number of gateways on the average network latency.

Figure 5 and Figure 7 show the comparison results of average network latency in the topology of Agis and Chinanet among the above algorithms when the number of gateways varied from 1–5. Obviously, the network latency presented a monotone decreasing tendency as the number of gateways increased. However, PKM always had better performance than SAA in Chinanet, while the opposite results occurred in Agis. It is noted that the gap of the performance between OEA and the other two algorithms gradually narrowed as the number of gateways increased.

### 5.2. Reliability Maximization with Latency Constraints

In the following, some comparison results of the joint placement problem between OEAJ, SACA, JPKM, and SAPKM are shown. The joint gateway and controller problem aims to maximize the network reliability with network latency constraints. Since satellite gateways and SDN controllers are deployed on certain switches separately, the latency plays a significant role in system performance. On the other hand, the controller-switch latency also affects the efficiency and effectiveness of the system performance, especially for supporting consistency. Thus, both controller-switch links and controller-gateway links determine the network reliability.

As mentioned above, the gateway-switch latency is defined as the network latency, which mainly depends on the placement of satellite gateways. Then, we investigated the joint placement problem using different algorithms, i.e., SACA, JPKM, and SAPKM, and made comparisons on the average network reliability, running time, and average/maximum controller-switch propagation latency. It is noted that JPKM is defined as the initial status of SAPKM, which means that JPKM only implements the first four steps in Algorithm 2.

**Figure 8:**
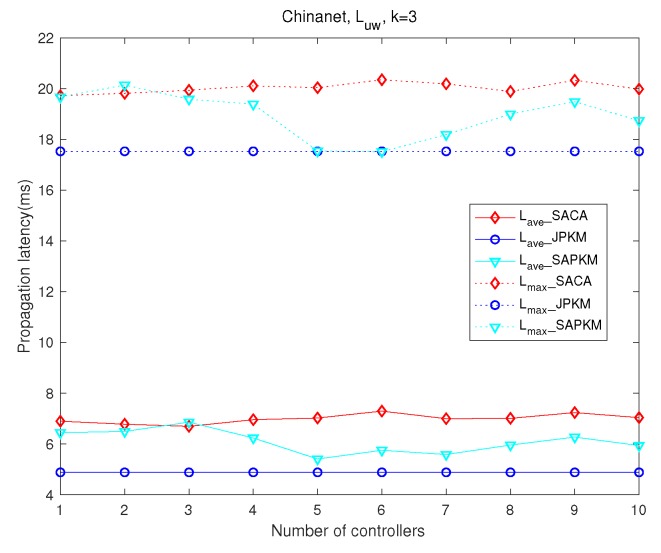
Comparisons of the overall network latency in Chinanet.

**Figure 9:**
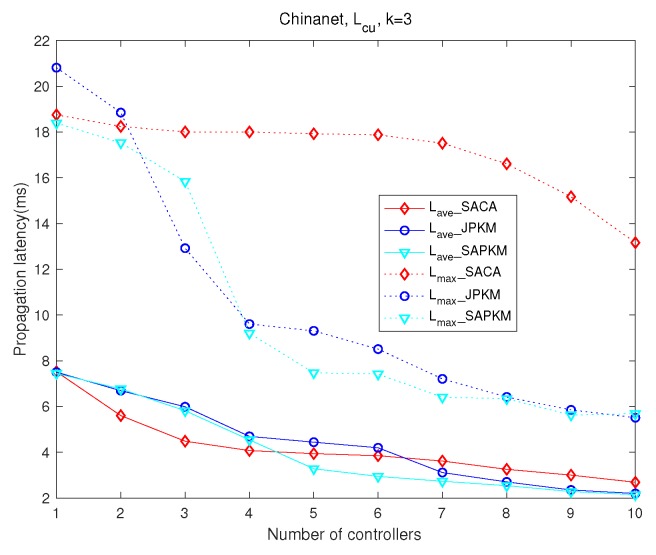
Comparisons of the overall controller-switch latency in Chinanet.

**Figure 10:**
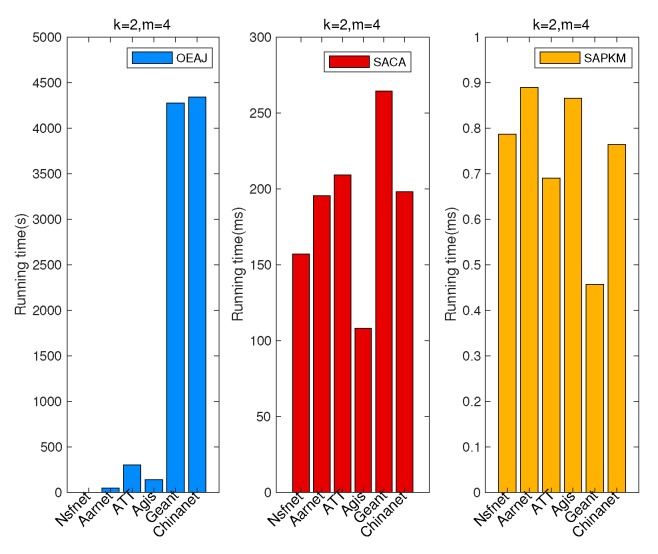
Comparisons of the overall running time.

**Figure 11:**
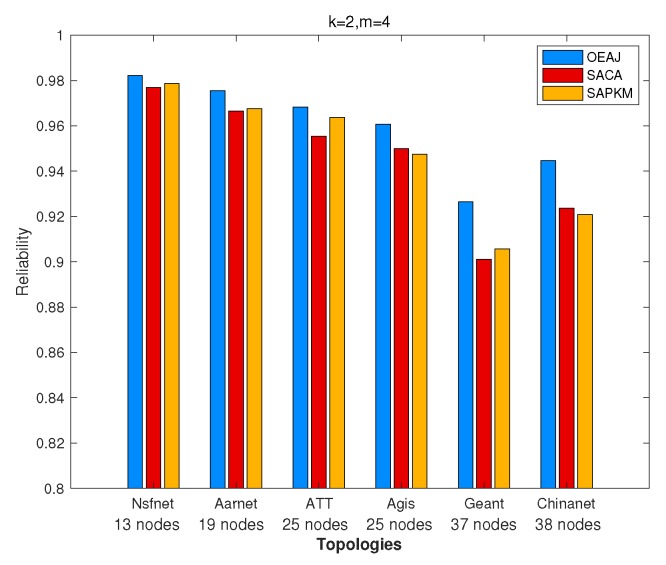
Comparisons of the overall average network reliability.

Figure 8 and Figure 9 analyze the effect of the number of controllers on network latency and controller-switch latency, respectively. Simulation results in the topology Chinanet are shown with three gateways deployed. Figure 10 and Figure 11 depict comparisons of both the overall running time and the overall average network reliability on the above topologies.

Figure 8 depicts the comparison of both maximum and average network latency in Chinanet, where the dotted lines represent the maximum network latency and the solid lines represent the average network latency. Since the purpose of solving the joint placement problem is to improve network reliability with network latency constraints, as long as the constraints are met, the proposed algorithm with better reliability performance is more advantageous than others. Apparently, JPKM had the best-settled latency performance among the joint placement algorithms when the number of controllers varied. Although JPKM outperformed SAPKM and SACA in Figure 8, one can observe that SAPKM had better performance than SACA on both maximum and average network latency. Since maximizing the network reliability adjusts the placement of both gateways and controllers, the latency performance of SACA and SAPKM fluctuated as the number of controller increased.

Figure 9 also shows a monotone decreasing trend of controller-switch propagation latency with an increasing number of controllers, where the dotted lines and the solid lines represent the maximum and the average controller-switch latency, respectively. Therein, SAPKM surpassed on average controller-switch latency when *m* was greater than four, while JPKM surpassed when *m* was larger than six. Besides, SACA always had the largest maximum controller-switch latency, which demonstrates the uneven distribution of controllers. Both JPKM and SAPKM ultimately obtained better average controller-switch propagation latency than that of SACA. Another virtue of both JPKM and SAPKM was the much lower maximum controller-switch propagation latency than that of SACA. The distribution of controller-switch latency demonstrated that the joint placement solutions derived from SAPKM were more reasonable than those of SACA.

Figure 10 demonstrates the comparison results of the overall running time in different topologies when the number of gateways was two and the number of controllers four. It is evident that SAPKM cost much less running time than SACA, with less than 1 ms to converge, while SACA cost hundreds of ms to converge, not to mention OEAJ. Furthermore, as shown in Figure 10, the running times of the given topologies varied from each other. It is noteworthy that the effect of the intrinsic properties of the given topologies on the running time of various algorithms is still unknown, which is one of our future research directions.

Figure 11 depicts the comparison results of the overall average network reliability in different topologies when the number of gateways was two and the number of controllers four. One can observe that SAPKM outperformed SACA in multiple topologies, i.e., Nsfnet, Aarnet, ATT, and Geant, while the opposite results occurred in Agis and Chinanet.

Thus, we further investigated whether SAPKM would have better average network reliability than that of SACA when the number of controllers increased. Owing to the enormous computing complexity of OEAJ, only simulation results in Agis are completely provided, and we mainly analyzed efficient algorithms on the performance and running time. To further evaluate the performance of SACA, JPKM, and SAPKM, we executed these algorithms on two different topologies, i.e., Agis and Chinanet, with k=3 and $L_{\mathrm{cst}}=10\;\mathrm{ms}$.

**Figure 12:**
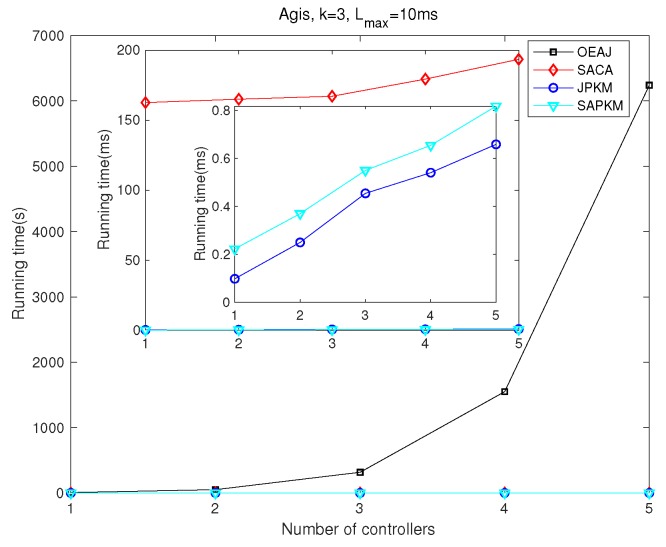
Comparisons of the overall running time in Agis.

**Figure 13:**
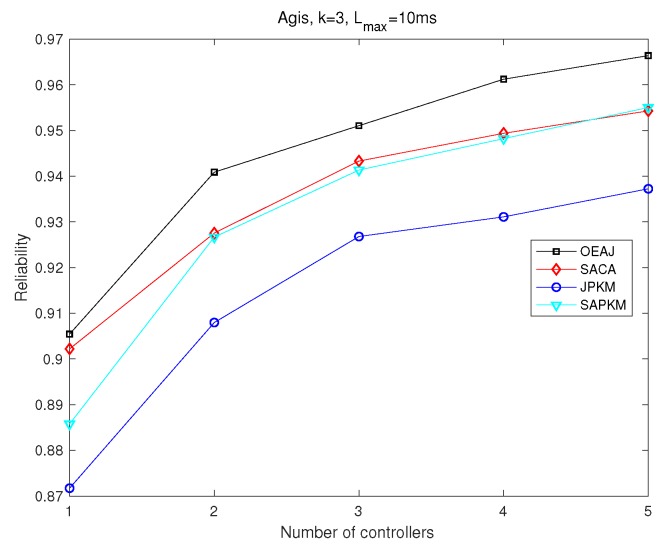
Comparisons of the overall average network reliability in Agis.

**Figure 14:**
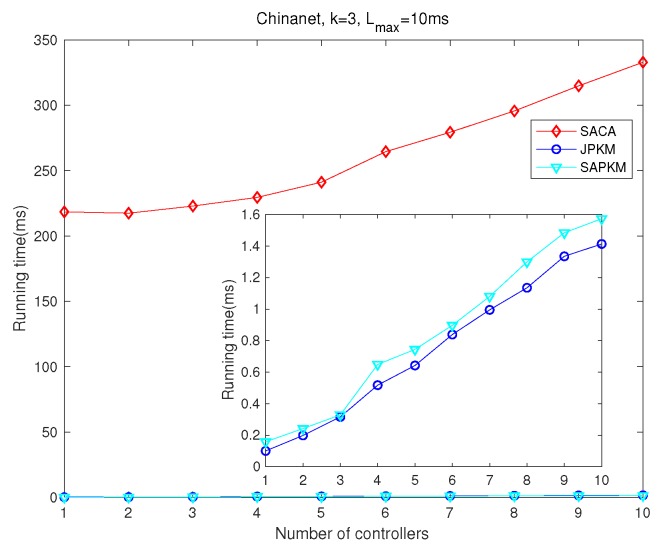
Comparisons of the overall running time in Chinanet.

**Figure 15:**
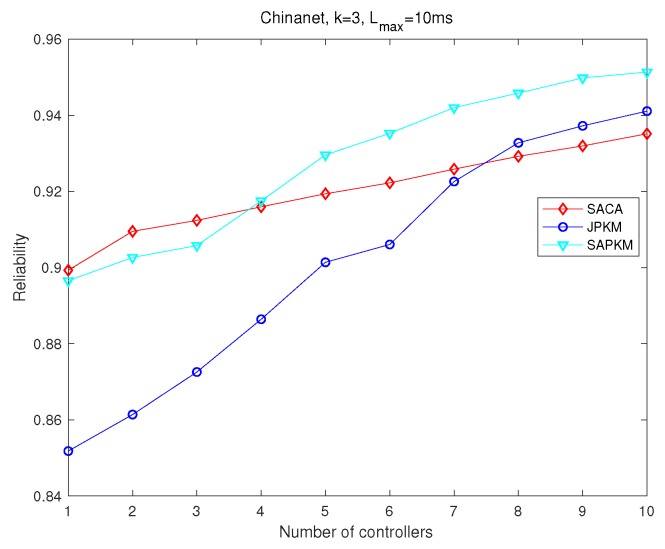
Comparisons of the overall average network reliability in Chinanet.

Simulation results depicted from Figure 12, Figure 13, Figure 14 and Figure 15 show the effect of the number of controllers on both the overall running time and the overall average network reliability in Agis and Chinanet, respectively. Figure 12 and Figure 13 were implemented on Agis with the number of controllers varied from 1–5, while Figure 14 and Figure 15 were implemented on Chinanet with the number of controllers varied from 1–10.

Figure 12 and Figure 14 show the effect of the number of controllers on the overall running time for Agis and Chinanet. Figure 12 shows the overall running time when the number of controllers varied from 1–5. Apparently, the computational complexity of both JPKM and SAPKM was more efficient than that of SACA and OEAJ, with about 1 ms for convergence. As *m* grew gradually, the running time of OEAJ increased exponentially, while that of the other algorithms increased slightly. Figure 14 implied that the running time of SACA, JPKM, and SAPKM also showed the same tendency in the topology of Chinanet with *m* increased from 1–10. Furthermore, we can observe the increment in running time between Chinanet and Agis, because there were 13 more nodes in Chinanet (38 nodes) than Agis (25 nodes).

Figure 13 and Figure 15 demonstrate the impact of the number of controllers on the overall average network reliability. Conspicuously, the network reliability had a monotone increasing tendency with a growing number of controllers. In Figure 13, OEAJ achieved an optimal solution, SACA and SAPKM obtained approximate solutions, and JPKM obtained the worst performance. What is more, SAPKM had almost the same network reliability as SACA did when *m* ranged from 2–4. SPAKM had a slightly better performance than that of SACA when *m* equaled five. In Figure 15, we mainly compared the performance among SACA, JPKM, and SAPKM. It is noteworthy that SAPKM had better network reliability than that of SACA when *m* was greater than or equal to four, and JPKM had the same tendency when *m* was greater than or equal to eight.

## 6. Conclusions

In this paper, we investigated the joint gateway and controller placement problem in SDN-enabled ISTNs. By adopting the network partition scheme, we proposed SAPKM to address the joint placement problem. Distinguished from previous works, we made full use of the intrinsic properties of the given topologies and selected the centroids of sub-domains as the initial sets of gateways and controllers. Owing to the efficiency of optimized initialization, we further decreased the number of redundant iterations in SAPKM, which dramatically shortened the running time and decreased the computing complexity of the algorithm. Experiments were performed on representative real network topologies with various structures and internal node densities to show the adaptiveness of the proposed algorithm. Simulation results also showed the superiority of SAPKM in network reliability, network propagation latency, and inter-plane latency due to the reasonable joint controller and gateway placement. Thus, the proposed algorithm is more applicable to large topologies for network reliability maximization and other metrics with multiple controllers and gateways for deployment. Besides, the merit of low complexity is also adaptive to the online joint placement problem in dynamic environments.

## Figures and Tables

**Table 1:** Performance metrics in pure SDN-enabled networks and SDN-enabled ISTNs.

Type of Network	Type of Path	Network Latency	Network Reliability	Inter-Plane Latency
Pure SDN-enabled networks	controller-switch		✓	✓
	gateway-switch	✓		
SDN-enabled ISTNs	controller-gateway		✓	
	controller-switch		✓	✓

**Table 2:** Notations and definitions.

Notation	Definition
G(V,E)	a physical network with node set V and link set E
V	V=U∪{s}
U	the set of terrestrial switch nodes
W	the set of satellite gateways
C	the set of SDN controllers
*s*	the satellite node
*w*	a satellite gateway in W
*c*	an SDN controller in C
*u*	a switch node in U
*k*	the number of satellite gateways
*m*	the number of SDN controllers
*n*	the number of switch nodes
$P_{u}$	failure probability of terrestrial nodes
$P_{e}$	failure probability of terrestrial links
$P_{e_{sw}}$	failure probability of the satellite link from *s* to *w*
$L_{uw}^{s}$	propagation latency from *u* to *s* via *w*
$L_{uw}$	propagation latency from *u* to *w*
$L_{sw}$	propagation latency from *w* to *s*
$L_{\mathrm{cst}}$	propagation latency constraint: maximum latency the terrestrial networks can tolerate
$L_{uc}$	propagation latency from *u* to *c*
$R_{uc}$	reliability of the shortest path from *u* to *c*
$R_{wc}^{s}$	reliability of *s* to *c* via *w* with the shortest terrestrial path

**Table 3:** Topology and failure probability settings.

Topology	Number of Nodes	Number of Links	$P_{u}$	$P_{e}$	$P_{e_{\mathrm{sw}}}$
Nsfnet	13	15	[0, 0.05]	[0, 0.02]	[0, 0.02]
Aarnet	19	24	[0, 0.05]	[0, 0.02]	[0, 0.02]
ATT	25	57	[0, 0.06]	[0, 0.04]	[0, 0.03]
Agis	25	30	[0, 0.06]	[0, 0.04]	[0, 0.03]
Geant	37	58	[0, 0.08]	[0, 0.08]	[0, 0.05]
Chinanet	38	62	[0, 0.08]	[0, 0.08]	[0, 0.05]

## Abbreviations

The following abbreviations are used in this manuscript:

3GPP	Third Generation Partnership Project
5G	Fifth Generation
CPP	Controller Placement Problem
GEO	Geostationary Orbit
gNB	gNodeBs
GPP	Gateway Placement Problem
ISTNs	Integrated Satellite-Terrestrial Networks
JPKM	Joint Partition-Based K-Means
NFV	Network Function Virtualization
OEA	Optimal Enumeration Algorithm
OEAJ	Optimal Enumeration Algorithm for the Joint placement problem
QoE	Quality off Experience
QoS	Quality of Service
PKM	Partition-Based K-Means
RAN	Radio Access Network
RNs	Relay Nodes
SAA	Simulated Annealing Algorithm
SACA	Simulated Annealing and Clustering hybrid Algorithm
SAPKM	Simulated Annealing Partition-based K-Means
SDN	Software-Defined Networking
